# The Lyme Disease Biobank: Characterization of 550 Patient and Control Samples from the East Coast and Upper Midwest of the United States

**DOI:** 10.1128/JCM.00032-20

**Published:** 2020-05-26

**Authors:** Elizabeth J. Horn, George Dempsey, Anna M. Schotthoefer, U. Lena Prisco, Matthew McArdle, Stephanie S. Gervasi, Marc Golightly, Cathy De Luca, Mel Evans, Bobbi S. Pritt, Elitza S. Theel, Radha Iyer, Dionysios Liveris, Guiqing Wang, Don Goldstein, Ira Schwartz

**Affiliations:** a Lyme Disease Biobank, Portland, Oregon, USA; b East Hampton Family Medicine, East Hampton, New York, USA; c Marshfield Clinic Research Institute, Marshfield, Wisconsin, USA; d Vineyard Center for Clinical Research, Martha’s Vineyard, Massachusetts, USA; e Department of Pathology, Stony Brook University, Stony Brook, New York, USA; f Department of Laboratory Medicine and Pathology, Mayo Clinic, Rochester, Minnesota, USA; g Department of Microbiology and Immunology, New York Medical College, Valhalla, New York, USA; University of Tennessee at Knoxville

**Keywords:** biobank, biorepository, Lyme disease, serology, diagnostics

## Abstract

Lyme disease (LD) is an increasing public health problem. Current laboratory testing is insensitive in early infection, the stage at which appropriate treatment is most effective in preventing disease sequelae. The Lyme Disease Biobank (LDB) collects samples from individuals with symptoms consistent with early LD presenting with or without erythema migrans (EM) or an annular, expanding skin lesion and uninfected individuals from areas of endemicity. Samples were collected from 550 participants (298 cases and 252 controls) according to institutional review board-approved protocols and shipped to a centralized biorepository. Testing was performed to confirm the presence of tick-borne pathogens by real-time PCR, and a subset of samples was tested for Borrelia burgdorferi by culture. Serology was performed on all samples using the CDC’s standard two-tiered testing algorithm (STTTA) for LD. LD diagnosis was supported by laboratory testing in 82 cases, including positive results by use of the STTTA, PCR, or culture or positive results by two enzyme-linked immunosorbent assays for cases presenting with EM lesion sizes of >5 cm. The remaining 216 cases had negative laboratory testing results. For the controls, 43 were positive by at least one of the tiers and 6 were positive by use of the STTTA. The results obtained with this collection highlight and reinforce the known limitations of serologic testing in early LD, with only 29% of individuals presenting with EM lesion sizes of >5 cm yielding a positive result using the STTTA. Aliquots of whole blood, serum, and urine from clinically characterized patients with and without LD are available to investigators in academia and industry for evaluation or development of novel diagnostic assays for LD, to continue to improve upon currently available methods.

## Abstract (precis)

Lyme disease (LD) is an increasing public health problem. Current laboratory testing is insensitive in early infection, the stage at which appropriate treatment is most effective in preventing disease sequelae. The Lyme Disease Biobank (LDB) collects samples from individuals with symptoms consistent with early LD presenting with or without erythema migrans (EM) or an annular, expanding skin lesion and uninfected individuals from areas of endemicity. Samples were collected from 550 participants (298 cases and 252 controls) according to institutional review board-approved protocols and shipped to a centralized biorepository.

## INTRODUCTION

The Lyme Disease Biobank (LDB) is a collection of human biological samples that facilitates research in Lyme disease (LD) and other tick-borne infections (TBI). The LDB was created in 2014 to provide well-characterized samples to investigators working to develop more accurate diagnostic tests for LD. In the United States, LD is caused primarily by the bacterium Borrelia burgdorferi *sensu stricto*, transmitted to a host through the blood meal of an infected *Ixodes* tick (1). Humans are incidental hosts and not part of the enzootic cycle. In the Upper Midwest, Borrelia mayonii is responsible for a small number of cases (2). Early LD is often characterized by erythema migrans (EM), an erythematous, expanding, skin lesion that develops at the site of the tick bite and that sometimes has a central clearing (3). While EM is a common manifestation of early LD, only 70 to 80% of individuals with early LD develop EM (4, 5); in the most recent U.S. Centers for Disease Control and Prevention (CDC) surveillance data from 2008 to 2015, 72.2% of individuals presented with EM (6). Even when present, EM may not have the classic bull’s-eye shape, which can confound a clinical diagnosis. Early LD can be accompanied by nonspecific, virus infection-like signs and symptoms, including headache, fever, chills, fatigue, myalgias, and arthralgias (3, 5). As the borreliae disseminate, multiple EM lesions may appear, as may 7th cranial nerve palsy, meningitis, or Lyme carditis. Late stages of LD include neuroborreliosis and Lyme arthritis (3, 5).

The diagnosis of early LD is based on clinical and epidemiological features and is sometimes supported by laboratory test results. For patients with EM lesions of >5 cm and a history compatible with tick exposure in an area of endemicity, a presumptive diagnosis of LD can be made and treatment can be initiated. Testing is not indicated for these patients, as the commonly used serologic methods would likely be negative due to a lack of detectable antibodies early in disease (3, 7). For the ∼30% of patients presenting without well-defined EM, an accurate diagnosis in the absence of positive laboratory test results is almost impossible.

Testing has traditionally been performed using a standard two-tiered testing algorithm (STTTA), which includes a first-tier enzyme immunoassay (EIA) or enzyme-linked immunosorbent assay (ELISA), and for those samples that are positive or equivocal (borderline), immunoblotting is performed. Using the interpretative algorithm published by the CDC, a positive immunoblotting result consists of at least 2 of 3 positive bands on the IgM immunoblot within 30 days of symptom onset or 5 to 10 bands on the IgG immunoblot at any time (8). More recently, the CDC endorsed a modified two-tiered testing algorithm (MTTTA). This approach still relies on a first-tier ELISA; however, in place of supplemental immunoblot testing, second-tier confirmatory testing is done using one or two other ELISAs with antigens different from those used in the first-tier ELISA (9). Several factors influence a positive serologic test result, including the duration of infection prior to sample collection, patient variability in the kinetics of the antibody response to an infectious agent, and the selection of appropriate antigenic targets (7).

The results of serologic tests may be negative in early LD, as there may not be sufficient time for the antibody response to develop, and results may be impacted based on which B. burgdorferi antigen is used in the first-tier assay. Direct detection methods, such as PCR and culture-based methods, have limitations, because B. burgdorferi is found at very low levels and only transiently in blood (3). Laboratory testing for B. burgdorferi, including limitations and future directions, has been reviewed previously (3, 7, 10–12). Insufficiencies in current testing methodologies complicate the accurate diagnosis of early LD, contribute to delays in diagnosis and treatment, and may result in additional sequelae. Aside from the recent MTTTA, there have been limited advances in LD diagnostic testing in the past 25 years, despite the growing public health concern. With an estimated 329,000 new cases each year in the United States, improved testing modalities are urgently needed (13).

The LDB was built out of the need to develop better diagnostics for LD. A summit of clinical, laboratory, nonprofit, and government stakeholders was convened in 2014 to discuss the current gaps in LD diagnostic testing, the types of samples needed to address the gaps, and potential protocols for sample collection. Well-characterized samples were available from the CDC Lyme Serum Repository (LSR) and the Study of LD Immunology and Clinical Events (SLICE) study (14, 15), but it was determined that these resources could not provide all the samples needed by the research community. An advisory committee of clinical and laboratory experts was formed, and a pilot study was launched on eastern Long Island, NY. Here, we present the characterization of 550 samples collected from individuals on the East Coast and Upper Midwest.

## MATERIALS AND METHODS

### Sample collection, processing, and storage.

Enrollment sites were selected based on their location in areas of endemicity (16) and their ability to identify and enroll patients with early LD. Contracts were individually negotiated and signed with each collecting site. Institutional review board (IRB) approval was obtained for each site through the LDB sponsor protocol (Advarra IRB protocol Pro00012408) or the site’s local IRB. For sites with a bilingual staff, consent forms were translated into Spanish, enabling the enrollment of Spanish-speaking participants. Individuals with signs or symptoms consistent with early LD were enrolled, including patients presenting with EM or an erythematous, annular, expanding skin lesion (annular lesion) and individuals presenting with signs or symptoms but without an EM/annular lesion and with a suspected tick exposure or tick bite. While individuals with annular lesions of ≤5 cm suspicious of LD were included, those with tick-bite reactions (e.g., a nonannular erythematous macule at the site of the tick bite) were excluded. Uninfected individuals from the same regions (controls from areas of endemicity [EC]) were also eligible to participate. Controls were defined as healthy individuals living in an area of endemicity with no history of LD or TBI. Inclusion and exclusion criteria are detailed in Table 1. Individuals enrolled in the type 1 group had EM/annular lesions, while individuals enrolled in the type 2 group had no skin manifestations.

**TABLE 1:** Inclusion and exclusion criteria for enrollment in the LDB^*a*^

Enrollment type	Inclusion criteria	Exclusion criteria
Type 1	Presents in region of endemicity	Immunocompromised^*b*^
	Physician identification/assessment	Antibiotics initiated >48 h^*c*^
	EM lesions of >5 cm (type 1A) or EM/annular expanding lesions of ≤5 cm (type 1B)	<10 yr of age
		Tick bite reaction without EM or expanding annular lesion
Type 2	Presents in region of endemicity	Immunocompromised
	Physician identification/assessment	History of CFS, rheumatologic disease, MS
	At least one of the following: headache, fatigue, fever, chills, or joint or muscular pain	Antibiotics initiated >48 h^*c*^ <10 yr of age
	Suspected tick exposure/tick bite	
EC	Residence in area where LD is endemic	Immunocompromised
		History of LD or other TBI
		<10 yr of age

a Abbreviations: LD, Lyme disease; EM, erythema migrans; CFS, chronic fatigue syndrome; MS, multiple sclerosis; EC, controls from areas of endemicity; TBI, tick-borne infection.

b Immunocompromised individuals, including individuals with HIV infection and individuals undergoing chemotherapy, were excluded. All participants were asked if they were taking immunosuppressive drugs.

c Antibiotics for LD had been initiated for >48 h at the time of study enrollment.

After informed consent was obtained, participants were assigned a unique identifier and samples (whole blood, serum, and urine) were collected according to IRB-approved protocols at the acute-phase blood draw. A case report form was used to collect information about signs and symptoms of early LD, about EM/annular lesions (if present, including a description of the features, duration, location, and measurements), whether antibiotics were being prescribed (the antibiotic name, dose, and duration), whether antibiotics were taken prior to the visit (the antibiotic name, dose, and duration), and whether any laboratory testing was ordered. Photographs of the EM/annular lesion (if present) were taken. Questions related to tick exposure were also asked, including if the participant reported or recalled a tick bite, the timing of the bite, the city and state where the bite occurred, information about attachment and engorgement, and the type of tick, if known. Both cases and controls were asked questions about current medications and specifically about immunosuppressive medications; their history of LD and TBI; their medical history, including a history of cancer or the presence of diseases of similar etiology (chronic fatigue syndrome, fibromyalgia, infectious mononucleosis, multiple sclerosis, rheumatoid arthritis, severe periodontitis, syphilis, or osteoarthritis); demographics; and city of permanent residence and length of residence there. Individuals ≥10 years of age were eligible to enroll.

Participants were enrolled based on clinical characteristics. Results of LD serology did not play a role in selecting potential participants, allowing for the enrollment of a broader patient population and minimizing reproofing of samples (i.e., providing samples for diagnostic development and test evaluation that had already been shown to be seropositive) (14). Cases were given an optional opportunity to provide second samples 2 to 3 months after the first visit (at a convalescent-phase blood draw). Updated clinical information was collected at the time of the convalescent-phase blood draw and at 6 and 12 months and included information on any ongoing signs or symptoms and current medications, including any additional antibiotics prescribed for LD. Participants were compensated with a $50 Amazon gift card or a $50 check for each blood draw, based on the compensation preference of the enrollment site.

The following sites participated: East Hampton, NY (2014 to 2018) (EH); Martha’s Vineyard, MA (2014 and 2015) (MV); and Marshfield, WI (2016 to 2018) (WI). Samples from Wisconsin were collected from Marshfield as well as clinics in Eau Claire, Lake Hallie, Minocqua, Wausau, and Weston. Each blood draw was ∼60 ml, with an additional 30 ml collected for culture experiments, described below. Samples were packed with refrigerated gel packs and shipped overnight by FedEx to a centralized biorepository (Precision for Medicine, Frederick, MD). On arrival, samples were processed and aliquoted as follows: serum separator tubes were spun according to the manufacturer’s instructions, and serum was aliquoted into 250-μl aliquots; whole blood collected in EDTA-containing tubes was aliquoted into 1- or 2-ml aliquots; urine was aliquoted into 1-ml aliquots. Several 5-ml aliquots of whole blood and urine were also created. All samples were transferred into screw-top cryovials and stored at −80°C.

### Sample testing.

Blind testing was performed on all samples from both cases and controls typically after the end of the season. Real-time PCR (RT-PCR) of whole blood from the acute-phase blood draw was performed to evaluate the samples for B. burgdorferi, Anaplasma phagocytophilum, Babesia microti, and Borrelia miyamotoi. Whole-blood samples from Wisconsin were also tested for Borrelia mayonii and Ehrlichia muris subsp. *eauclairensis*, two tick-borne bacteria that have been reported only from the Upper Midwest. RT-PCR of whole blood was performed at New York Medical College (NYMC) or Mayo Clinic (MC) as follows: at NYMC for samples collected from 2014 to 2017 at EH and in 2015 and 2016 from MV and at MC for samples collected from 2016 to 2018 in WI and in 2018 at EH. Specimens were tested at NYMC (17, 18) and MC (2, 19, 20) using previously published protocols.

A subset of samples was tested by culture followed by PCR of the culture fluid for B. burgdorferi, essentially as described previously with minor modifications (21). All culture experiments were conducted at NYMC (2014 to 2016). Several methods were explored to send samples to NYMC for processing. In 2014, 30 ml of whole blood collected in EDTA-containing tubes was shipped overnight with refrigerated gel packs. Upon arrival, plasma was isolated from the whole blood, and Barbour-Stoenner-Kelly (BSK) medium was inoculated. This was also done for a subset of samples in 2016. In 2015, however, plasma was isolated and BSK medium was inoculated at the site, with flasks containing inoculated medium being shipped overnight at ambient temperature.

Serologic testing for LD using STTTA was performed on sera from all samples (acute- and convalescent-phase blood, when available). Testing was not reflexive (e.g., immunoblotting was performed on all samples regardless of the first-tier test results). Serologic testing was performed at Stony Brook University (SB) or MC, as follows: at SB for samples collected from 2014 to 2017 at EH and in 2015 and 2016 at MV and at MC for samples collected from 2016 to 2018 in WI and in 2018 at EH. In addition, samples collected in EH in 2018 and samples collected in WI in 2017 and 2018 were tested at SB with the C6 peptide ELISA (Oxford Immunotec, Marlborough, MA). Testing at SB included a laboratory-developed ELISA based on whole-cell lysate from B. burgdorferi, the C6 peptide ELISA, and laboratory-developed anti-B. burgdorferi IgM and IgG immunoblotting. MC used the following Food and Drug Administration (FDA)-cleared assays for anti-B. burgdorferi antibody testing: for first-tier testing, the C6 peptide ELISA was used in 2016 and the VlsE/pepC10 IgM/IgG ELISA (Zeus Scientific, Raritan, NJ) was used in 2017 and 2018, and IgM and IgG immunoblot testing was done using ViraStripe blots (Viramed; Biotech AG, Germany). All testing was performed according to the manufacturers’ instructions. All samples were tested by two ELISAs, one of which was the C6 peptide ELISA, except for samples tested in 2016 at MC, which were tested only by the C6 peptide ELISA. The results of all immunoblot assays were interpreted using CDC criteria. A sample was considered two-tier testing positive by the CDC criteria (STTTA) if at least one of the ELISAs was positive or equivocal and at least 2 of 3 bands were present on the IgM blot in patients with less than 30 days of signs/symptoms and/or if 5 to 10 bands were present on the IgG blot at any time (8). For IgM, a minimum of 2 of the 3 bands of 23, 39, and 41 kDa had to be present, and for IgG, a minimum of 5 of the 10 bands of 18, 23, 28, 30, 39, 41, 45, 58, 66, and 93 kDa had to be present. Participants were considered to have seroconverted if the IgG immunoblot assay result became positive at the convalescent-phase blood draw following an initial negative result at the acute-phase blood draw.

### Laboratory accreditation.

All RT-PCR assays and serologic testing were performed in College of American Pathologists (CAP)-accredited and Clinical Laboratory Improvement Amendments (CLIA)-certified clinical laboratories with experience in Lyme disease testing. Precision for Medicine is also accredited by CAP and CLIA certified.

### Statistics.

The differences in the ages of the participants among the enrollment groups (participants in the type 1 or type 2 group and EC) and collection sites (EH, MV, WI) were examined using one-way analysis of variance. The differences in the ages of enrolled male and female participants were also separately statistically investigated by group or site using one-way analysis of variance. In all cases, assumptions about the normality of residuals and the homogeneity of variance were checked, and the parametric test was used if these assumptions were met. When the overall among-group effect (omnibus test) was significant, *post hoc* pairwise comparisons were performed with a Tukey honestly significant difference test. Statistical tests were performed in R (version 3.6.1), including by use of the car package (22, 23).

Differences in the proportions of individuals who tested positive for LD according to STTTA were examined between and among enrollment groups with nonparametric tests for the equality of proportions with the Yates continuity correction for small sample size. Two-tailed tests were considered in all cases, and when more than 2 groups were compared, differences were investigated with an overall, omnibus test of the equality of proportions as well as individual pairwise comparisons between groups. The significance of pairwise comparisons was evaluated with an adjusted alpha value of 0.016 to reduce the risk of a type 1 error. Statistical tests were performed in R (version 3.6.1) software (22).

### Sample categorization.

Once the test results were received, all samples collected from enrolled patients with signs or symptoms of early LD (i.e., cases) were classified into the following categories by the LDB: laboratory testing-confirmed LD, which was a positive result by use of the STTTA, a positive PCR result for B. burgdorferi, positive results by culture/PCR of the culture media, or positive results by two ELISAs for individuals with EM lesion sizes of >5 cm; probable LD, which was an EM lesion size of >5 cm, a negative result by use of the STTTA, and a negative result by PCR for B. burgdorferi; suspected LD, which was an EM/annular lesion size of ≤5 cm, a negative result by use of the STTTA, and a negative result by PCR for B. burgdorferi; and symptomatic no lesion (SNL), which was the presence of clinical signs or symptoms without EM/annular lesions, a negative result by use of the STTTA, and a negative result by PCR for B. burgdorferi.

All enrolled patients presented with clinical signs or constitutional symptoms consistent with early LD. The group with laboratory testing-confirmed LD included patients for whom the diagnosis was confirmed by laboratory testing, including STTTA, PCR for B. burgdorferi, and/or culture; samples from individuals with positive results by two ELISAs and EM lesion sizes of >5 cm were also included (24). This group with laboratory testing-confirmed LD included patients who were enrolled with EM as well as those without EM/annular lesions. The probable and suspected LD groups included patients with a clinical diagnosis consistent with early LD, based on the presence of EM (lesion size, >5 cm; probable LD) or EM/annular lesions (lesion size, ≤5 cm; suspected LD), but with negative laboratory test results. For the purposes of this study, we categorized samples that were negative on laboratory testing and EM lesion sizes of >5 cm as probable LD and samples that had negative laboratory testing results and an EM/annular lesion size of ≤5 cm as suspected LD (note that these are different from the definitions used for CDC surveillance criteria) (25). All samples characterized as probable LD and suspected LD were negative by use of the STTTA; however, they may have had a positive serologic result on either first-tier or second-tier testing. The SNL group included patients without EM/annular lesions that were suspected to have early LD due to signs/symptoms and exposure history by the enrolling provider but that were negative by current laboratory testing methods. As with the groups of probable and suspected LD, these samples were STTTA negative but could have had a positive serologic test result by one of the individual tests within the algorithm. Individuals enrolled as controls were classified as controls from areas of endemicity (the EC group) with negative results by both tiers or EC with a positive serology, when the result of serologic testing was positive on testing by at least one tier.

### Applying for samples.

Aliquots of whole blood, serum, and urine are available to investigators through a peer-reviewed application process. Briefly, applications for samples are submitted to LDB and reviewed by the LDB principal investigator and then by an ad hoc committee consisting of two individuals with appropriate subject matter expertise to review the proposal. Individual peer reviewers are selected from a list of ∼40 individuals with expertise in LD and TBI. Applications that exhibit technical merit, the potential to advance LD and other TBI diagnostics, and the likelihood to increase knowledge of LD and other TBI and that are in alignment with LDB goals and objectives are recommended for approval. The LDB board of directors makes the final determination as to which applicants will be approved to receive samples. A sample and data access agreement that includes a material transfer agreement requiring data sharing back to LDB is negotiated with each sample user. LDB also makes available panels for which the results are known for projects earlier in the process of development. These requests are also approved by the LDB board of directors but do not undergo peer review. Preliminary data from panels for which the results are known can then be used to support applications for additional samples.

### Sample distribution.

Sample pulls are customized for each approved investigator, based on the research question being asked, and can be limited to specific categories (i.e., investigators may request samples only from participants with laboratory testing-confirmed LD and controls or samples from participants with laboratory testing-confirmed LD, probable LD, and controls, etc., based on the stage of the project). A random-number generator is used to select the specific samples from each category that will be sent to the investigator. All samples are accompanied by robust clinical information and test results. Samples are typically provided so that that the investigators are initially blind to their results.

### Data availability.

For more information about receiving samples, please contact info@lymebiobank.org.

## RESULTS

### Enrollment.

Samples were collected prospectively at three community sites: East Hampton, NY (EH); Martha’s Vineyard, MA (MV); and Marshfield, WI (WI). Each site enrolled individuals presenting with signs or symptoms consistent with early LD, with or without EM or annular lesions, and EC (Table 1). Individuals enrolled as cases could have had previous exposure to LD or TBI in prior years that resolved. Participants in the type 2 group included individuals who were symptomatic with suspected tick exposure. EC were generally healthy without previous known LD or TBI. The majority of cases (82%) were antibiotic naive at the time of enrollment; however, individuals could enroll within 48 h of starting antibiotics for LD.

For each collection season except the pilot, enrollment opened on 15 April, with peak enrollment occurring in June and July, a time consistent with nymphal tick activity, and continuing through the late fall, a time consistent with adult tick activity (26). Enrollment at the acute-phase visit was completed by mid-December, with convalescent-phase blood draws occurring 2 to 3 months after the initial draw. In 5 seasons, 550 individuals were enrolled, with 65% of the participants being enrolled on the East Coast and 35% being enrolled in the Upper Midwest (Table 2). For individuals enrolled in the type 1 group, 41% had no signs/symptoms outside of the EM/annular lesion, 43% had more than one sign/symptom, and 14% had more than three. Among the participants in the type 2 group, 84% had more than one sign/symptom and 45% had more than three. For this analysis, the type 1 group was further divided into individuals with EM lesions of >5 cm (type 1A) or EM/annular lesions of ≤5 cm (type 1B). Three-quarters (*n* = 146) of those enrolled in the type 1 group presented with physician-diagnosed EM lesions of >5 cm, including 82% enrolled at EH, 73% enrolled at MV, and 63% enrolled at WI, consistent with a clinical diagnosis of LD (3). For participants who agreed to be recontacted, 160 (54% of cases) returned for a convalescent-phase blood draw at EH or WI.

**TABLE 2:** Characteristics of participants enrolled, 2014 to 2018^*a*^

Characteristic	Value for participants in the following group:			
	Type 1 (*n* = 196)	Type 2 (*n* = 102)	EC (*n* = 252)	Total (*n* = 550)
No. of participants by yr [site(s)]				
2014 (EH)	10	12	22	44
2015 (EH, MV)	27	16	33	76
2016 (EH, MV, WI)	47	29	71	147
2017 (EH, WI)	65	25	78	168
2018 (EH, WI)	47	20	48	115
				
No. of participants by site (type)				
EH total (type 1A, type 1B)	123 (101, 22)	59	136	318
MV total (type 1A, type 1B)	11 (8, 3)	11	18	40
WI total (type 1A, type 1B)	62 (39, 23)	32	98	192
				
No. (%) of participants with the following signs and symptoms:				
Headache	70 (36)	81 (79)	NA	
Fatigue	96 (49)	80 (78)	NA	
Fever	36 (18)	45 (44)	NA	
Chills	55 (28)	45 (44)	NA	
Joint/muscle pain	88 (45)	78 (76)	NA	

a Abbreviations and definitions: EH, East Hampton, NY; MV, Martha’s Vineyard, MA; WI, Marshfield, WI; type 1A, EM lesion size of >5 cm; type 1B, EM/annular lesion size of ≤5 cm; NA, not applicable.

### Demographics.

A larger percentage of men were enrolled as cases (59% in the type 1 group, 63% in the type 2 group). More women enrolled as EC (67%), particularly in the Upper Midwest (Table 3). Nearly all were adults. The mean ages for men and women in the cohort were 51.2 and 55.1 years, respectively, for participants in the type 1 group; 53.5 and 48.0 years, respectively, for participants in the type 2 group; and 48.5 and 45.1 years, respectively, for EC (Table 3). The ages of the participants were not statistically significantly different across the enrollment sites (*P* = 0.503). However, the ages of the participants differed across enrollment groups (*P* = 0.0003), and *post hoc* tests revealed that the participants were, on average, younger in the EC group than in the type 1 group (*P* = 0.0003) or the type 2 group (*P* = 0.033). There were no statistically significant differences in age between the type 1 and the type 2 groups. The majority of participants were Caucasian (77% among participants in the type 1 group, 78% among participants in the type 2 group, 85% among EC), followed by Hispanic or Latino (22% among participants in the type 1 group, 20% among participants in the type 2 group, 11% among EC) (Table 4).

**TABLE 3:** Demographics by age and sex at each site

Characteristic	Site^*a*^	Value for participants in the following group:		
		Type 1 (*n* = 196)	Type 2 (*n* = 102)	EC (*n* = 252)
No. (%) of participants				
Male	EH	74 (60)	37 (63)	65 (48)
	MV	3 (27)	6 (55)	3 (17)
	WI	39 (63)	21 (66)	16 (16)
	Total	116 (59)	64 (63)	84 (33)
Female	EH	49 (40)	22 (37)	71 (52)
	MV	8 (73)	5 (45)	15 (83)
	WI	23 (37)	11 (34)	82 (84)
	Total	80 (41)	38 (37)	168 (67)
Mean age (yr) of participants				
Male	EH	48.1	50.1	49.6
	MV	64.3	49.9	41.3
	WI	56.1	60.5	45.6
	Total	51.2	53.5	48.5
Female	EH	54.3	47.1	48.0
	MV	48.4	51.3	40.3
	WI	59.0	48.5	43.4
	Total	55.1	48.0	45.1

a Abbreviations: EH, East Hampton, NY; MV, Martha’s Vineyard, MA; WI, Marshfield, WI, which includes samples from Eau Claire, Lake Hallie, Marshfield, Minocqua, Wausau, and Weston.

**TABLE 4:** Demographics by race and ethnicity across all sites

Race or ethnicity	No. (%) of participants in the following group:		
	Type 1 (*n* = 196)	Type 2 (*n* = 102)	EC (*n* = 252)
American Indian or Alaska Native	0 (0)	1 (1)	0 (0)
Asian	1 (0.5)	0 (0)	7 (2.8)
Black or African American	2 (1)	1 (1)	1 (0.4)
Hispanic or Latino^*a*^	43 (21.9)	20 (19.6)	27 (10.7)
Native Hawaiian or Pacific Islander	0 (0)	0 (0)	1 (0.4)
White	150 (76.5)	80 (78.4)	213 (84.5)
Multiple races	0 (0)	0 (0)	1 (0.4)
Participants refused to identify	0 (0)	0 (0)	2 (0.8)

a All Hispanic or Latino participants were enrolled at East Hampton, except for 1 participant with EC enrolled at Wisconsin.

### Sample testing.

Testing results for participants in the type 1 group were divided into two groups: type 1A, in which the EM lesions were >5 cm, or type 1B, in which EM/annular lesions were ≤5 cm.

### Culture.

In collaboration with NYMC, a subset of samples was tested by culture followed by PCR of the culture media (21). This included all samples collected in the first season and a smaller number of samples from patients presenting with EM or EC during the following 2 seasons (19 samples from participants in the type 1A group, 6 samples from participants in the type 1B group, 20 samples from participants in the type 2 group, and 26 samples from EC, with 32% of the participants in the type 1A group, 33% of the participants in the type 1B group, and 20% of participants in the type 2 group having received antibiotics for <48 h of at the time of sample collection). B. burgdorferi isolates were cultivated from 2 samples from participants in the type 1A group. Upon sequencing, one of these isolates was determined to be OspC type A and sequence type 1 (ST1), making it equivalent to strain B31, and the other isolate was OspC type D and ST38, a rarer isolate. When PCR was performed on the culture media, 10 samples were positive; these included the samples with the 2 isolates mentioned above and were from 8 participants in the type 1A group, 1 participant in the type 1B group, and 1 EC. The positive result for the EC may have been due to contamination, sample mislabeling, or an asymptomatic infection.

### RT-PCR.

Only 4 samples RT-PCR positive for B. burgdorferi were identified; 3 samples were from participants in the type 1A group and 1 sample was from a participant in the type 2 group. Testing for other TBI was performed, with B. microti *and* A. phagocytophilum being selected due to their prevalence on the East Coast and Upper Midwest, respectively. Evidence of B. microti infection was found in 7% of samples from participants in the type 1A group and 2% of samples from participants in the type 2 group, while evidence of A. phagocytophilum infection was less common (<1% of samples from participants in the type 1A group and 2% of samples from participants in the type 2 group). Samples were also tested for B. miyamotoi, and samples from participants enrolled from WI were tested for B. mayonii and Ehrlichia muris subsp. *eauclairensis*, with no samples having positive RT-PCR results for these pathogens.

### Serology.

Serologic testing results for the acute- and convalescent-phase blood draws are shown in Table 5. Participants presenting with EM lesions of >5 cm were more likely to be positive by any serologic test. For the acute-phase blood draw, a positive ELISA result was found for 38 to 43% of participants in the type 1A group, 17 to 35% of participants in the type 1B group, 15 to 27% of participants in the type 2 group, and 5 to 11% of EC (ranges represent the percentage of participants positive using the different first-tier tests). For the immunoblot assays, more cases were positive for IgM than for IgG (as would be expected for early LD). However, there were no statistically significant differences between the participants in the type 1 and type 2 groups or between participants in the type 1A and type 1B groups for IgM or IgG positivity. When the STTTA was applied, the results for 29% of participants in the type 1A group, 10% of participants in the type 1B group, 12% of participants in the type 2 group, and 2% of EC were interpreted as positive. Cases were enrolled within 30 days of sign or symptom onset, making the IgM results relevant to determining two-tier testing positivity at the acute-phase blood draw. The proportion of two-tier testing-positive samples was significantly different between the type 1 and type 2 groups (*P* = 0.01) and between the type 1A and type 1B groups (*P* = 0.015). The results for the controls from areas of endemicity were not considered in the statistical analysis comparing proportions. For participants with a clinical diagnosis (type 1A), 71% were negative by use of the STTTA. At the convalescent-phase blood draw, the rate of IgG seropositivity remained low, and seroconversion, defined as IgG seropositivity at the convalescent-phase blood draw when the participant was negative at the acute-phase blood draw, was <5% overall.

**TABLE 5:** Serology results at acute- and convalescent-phase blood draws^*a*^

Testing	No. of participants or no. of participants with the indicated result/total no. tested (%)			
	Type 1A	Type 1B	Type 2 (all)	EC (all)
Acute-phase visit	148	48	102	252
Whole-cell lysate ELISA +	35/82 (43)	6/24 (25)	10/58 (17)	15/133 (11)
C6 peptide ELISA +	62/148 (42)	8/48 (17)	15/102 (15)	14/252 (5)
VlsE/pepC10 ELISA +	23/60 (38)	7/20 (35)	9/33 (27)	14/97 (14)
IgM immunoblotting +	44/148 (30)	5/48 (10)	17/102 (17)	13/252 (5)
IgG immunoblotting +	12/148 (8)	1/48 (2)	6/102 (6)	4/252 (2)
Two-tier testing + (STTTA)^*b*^	43/148 (29)	5/48 (10)	12/102 (12)	6/252 (2)
Convalescent-phase visit	83	31	46	NA
Whole-cell lysate ELISA +	18/38 (47)	2/12 (17)	3/20 (15)	NA
C6 peptide ELISA +	42/83 (49)	4/31 (13)	8/46 (17)	NA
VlsE/pepC10 ELISA +	15/43 (35)	4/15 (27)	8/18 (44)	NA
IgM immunoblotting +	27/83 (33)	4/31 (13)	7/46 (15)	NA
IgG immunoblotting +	6/83 (7)	1/31 (3)	5/46 (11)	NA
Seroconversion^*c*^	3/83 (4)	1/31 (3)	3/46 (7)	NA

a Abbreviations and definitions: +, positive; STTTA, standard two-tiered testing algorithm; NA, not applicable; type 1A, EM lesion size of >5 cm; type 1B, EM/annular lesion size of ≤5 cm. For participants enrolled as type 1 and type 2, positive whole-cell lysate, C6 peptide, and VlsE/pepC10 ELISA results include both positive and equivocal results. Serologic testing was performed at Mayo Clinic (MC) or Stony Brook University (SB).

b An STTTA positive result is a positive or equivocal whole-cell lysate ELISA, C6 peptide ELISA, or VlsE/pepC10 ELISA result followed by a positive IgM immunoblotting or IgG immunoblotting result using the STTTA result at the acute-phase blood draw.

c Seroconversion was indicated by a positive IgG immunoblotting result at the convalescent-phase blood draw following a negative IgG immunoblotting result at the acute-phase blood draw.

### Categorization.

The results described above were used to categorize the samples in the LDB (Table 6). Of those enrolled as type 1, 35% (31% type 1A and 4% type 1B) were classified as having laboratory testing-confirmed LD, 44% were classified as having probable LD, and 21% were classified as having suspected LD. Of those enrolled as type 2, 14% were classified were classified as having laboratory testing-confirmed LD and 86% were classified as having SNL. The majority of EC (83%) were negative by all serologic tests.

**TABLE 6:** Categorization of LDB samples^*a*^

Classification^*b*^	Criteria	No. of participants with the indicated characteristics/total no. tested (%)
Laboratory-confirmed LD	+ STTTA result, + PCR result, + culture/PCR of the culture media, or 2 + ELISAs with EM of >5 cm	82/298 (27)
Probable LD	EM lesions of >5 cm, − STTTA result, and – PCR result	87/298 (29)
Suspected LD	EM/annular lesion size of ≤5 cm, − STTTA result, and − PCR result	41/298 (14)
SNL	Symptomatic, − STTTA result, and − PCR result	88/298 (30)
EC (− serology)	− by serology (all serologic tests)	209/252 (83)
EC (+ serology)^+^	+ by serology (at least 1 + serologic test)	43/252 (17)

a +, positive; −, negative; STTTA, standard two-tiered testing algorithm; SNL, symptomatic no lesion; EC (+ serology)^+^, a minimum of 1 positive test result by whole-cell lysate, C6 peptide, or VlsE/pepC10 ELISA, IgM immunoblotting, or IgG immunoblotting.

b For the laboratory testing-confirmed category, samples were categorized by positive test results with serology by use of the STTTA (*n *= 60), positive results by 2 ELISAs with EM lesion size of >5 cm (*n *= 11), or IgG seroconversion at the convalescent-phase blood draw (*n *= 7). Tests for direct detection confirmation included culture (*n *= 2), PCR of culture medium (*n *= 9), or RT-PCR of whole blood (*n *= 4). The results for the samples could be confirmed with multiple tests. Participants with probable LD, suspected LD, or SNL had signs or symptoms consistent with early, acute LD that could include skin manifestations (EM/annular lesion), headache, fatigue, fever, chills, or joint or muscle pain but did not meet the criteria for laboratory testing-confirmed LD.

## DISCUSSION

LDB provides well-characterized samples to investigators studying LD and TBI. As noted by the CDC LSR, large, well-characterized sample sets can benefit medical providers, test developers, and the public at risk for LD (14). Well-characterized samples are vital for diagnostic test development and validation. It is critical that sample users know what criteria were used to enroll participants; how the samples were collected, processed, and stored; and what clinical and testing data are available. Additional comparative benefits are realized when test developers creating new tests and optimizing current tests are using the same well-characterized samples.

The LDB complements the CDC LSR and the SLICE study. The CDC LSR provides serum only and has samples from individuals with additional clinical presentations of LD (Lyme carditis, late Lyme arthritis) and diseases with an etiology similar to that of LD (14). The SLICE study has multiple sample types collected over the course of a year but is limited to samples from individuals presenting with EM (15). LDB includes serum, whole blood, and urine, and individuals were enrolled based on clinical criteria for signs or symptoms consistent with early LD.

Samples from participants that were laboratory testing confirmed (*n* = 82) were more likely to be enrolled as type 1 (83%) and were more likely to present with EM lesions of >5 cm (74%). However, 26% of participants with laboratory testing-confirmed LD did not present with EM lesions of >5 cm, including 9% enrolled as type 1B (having EM/annular lesions of ≤5 cm) and 17% enrolled as type 2 without skin manifestations. This reinforces that LD does occur, although less frequently, in individuals who do not have EM or that the EM/annular lesion may not have reached >5 cm at the time that the individual presents to the medical provider. Others have noted that patients with LD do not always present with EM and that many LD-associated lesions do not have central clearing (27, 28). In a small study of samples from patients with PCR-confirmed LD, 10 of 14 patients presented with nonclassical EM and only 4 presented with the classic presentation of EM with central clearing (28). A lack of provider awareness of the absence of EM or nonclassical EM presentations can lead to underdiagnosis and delayed treatment, highlighting the need for better diagnostics for early LD.

Only 16% of the laboratory testing-confirmed LD cases were confirmed by direct detection methods, and only 6 samples (7%) were confirmed by both serology and direct detection methods (i.e., culture/PCR of culture media). The gold standard test in microbiology is the ability to culture the pathogen. Spirochetes are challenging to culture; however, B. burgdorferi has been cultured successfully from skin biopsy specimens of EM lesions, whole blood, and serum, with the highest success rates being from EM skin biopsy specimens (3). Positive culture rates were low and were obtained for only 11% of samples from participants with laboratory testing-confirmed LD. Collection of skin biopsy specimens from EM lesions would have likely improved the positive culture rates; however, such invasive testing is not routinely performed. In addition, the extended time needed to culture is a disadvantage to the use of culture in the clinical setting. The inclusion of patients who had started antibiotics may have decreased the ability to culture the bacteria (29, 30), as may have delays in sample processing due to shipping. While inoculation of culture media was attempted immediately at one site, culture rates did not improve. The logistics of sample processing likely contributed to culture rates lower than those previously reported (31–33). PCR of blood for B. burgdorferi is often insensitive in early disease, as was seen in this cohort, with only 5% of the samples from participants with laboratory testing-confirmed LD being PCR positive. None of the PCR-positive samples were positive by use of the STTTA, although one individual was found to have seroconverted at the convalescent-phase blood draw.

The majority of laboratory testing-confirmed LD cases were confirmed by use of the STTTA (73%). Notably, the rate of positivity by use of the STTTA, which was positive for 29% of patients classified as type 1A, was lower than that in prior published studies. In one study, the sensitivity of the STTTA was 38% in patients with EM during the acute phase (34), whereas in the CDC LSR, 40% of samples from individuals with EM lesions of >5 cm were two-tier testing positive (14). We hypothesize that people in areas where LD is highly endemic may be highly motivated to seek medical care at the first signs/symptoms of LD. The low seropositivity rates could be due to early identification and intervention at these sites. In a study of 46 patients with culture-confirmed EM, seropositivity at baseline correlated with disease duration prior to treatment (35). For those enrolled as type 1, 41% had no signs/symptoms outside of EM/annular lesions, which may represent patients seeing the provider earlier in the course of infection, prior to the development of additional signs/symptoms. For those presenting without EM/annular lesions, other organisms, such as spirochetes causing relapsing fever, could overlap B. burgdorferi in these regions of endemicity, although we did not detect B. miyamotoi in these samples by RT-PCR. It is of note that 17% of EC had at least one positive serologic test result, with 2% being positive by use of the STTTA. The low level of STTTA positivity in EC supports the specificity of the tests and provides confidence that the testing results for cases represent positive results for LD. These levels of seropositivity are in alignment with testing of controls from areas of endemicity in other studies, including the CDC LSR, where 2% of controls were STTTA positive (14).

IgG seroconversion rates in the LDB were also much lower than those in earlier published studies (35, 36). It is of note that the vast majority of cases were treated with doxycycline upon enrollment. Early antibiotic treatment impacts B-cell expansion and the seroconversion rate. In a study of the serologic responses in patients with culture-confirmed EM, the rate of seroconversion correlated with disease duration and/or dissemination prior to treatment (35). The class of antibiotic may also influence or mute the immune response. In early studies, participants were treated with antibiotics other than doxycycline, such as azithromycin (36). In a study of 104 patients with EM who were treated with doxycycline, IgG seroconversion was infrequent at the second draw (15). The timing of the convalescent-phase blood draw also impacts the performance of serologic assays. For example, the CDC LSR convalescent-phase blood draws occurred between 10 and 35 days after the initial diagnosis and initiation of antibiotic treatment, whereas LDB’s convalescent-phase blood draw occurred 2 to 3 months after the acute-phase blood draw. At this later time, only IgG can be used to determine seroconversion.

As with any biorepository, there are limitations. The LDB focused on enrolling participants presenting with signs or symptoms consistent with early LD, with or without EM/annular lesions. While this provides broader inclusion criteria, it is possible that some of the participants enrolled as type 2 did not have LD. These 550 samples were only from individuals from the East Coast and Upper Midwest, and samples from individuals infected on the West Coast were not represented. However, efforts are ongoing to collect samples from individuals infected with B. burgdorferi in California. The LDB initially did not include samples from patients with other stages of LD but has recently added to the collection samples from participants with neuroborreliosis and Lyme carditis.
